# A Pilot Study of IL2 in Drug-Resistant Idiopathic Nephrotic Syndrome

**DOI:** 10.1371/journal.pone.0138343

**Published:** 2015-09-28

**Authors:** Alice Bonanni, Roberta Bertelli, Roberta Rossi, Maurizio Bruschi, Armando Di Donato, Pietro Ravani, Gian Marco Ghiggeri

**Affiliations:** 1 Division of Nephrology, Dialysis, Transplantation and Laboratory on Physiopathology of Uremia, Giannina Gaslini Children Hospital, Genoa, Italy; 2 Division of Nephrology, University of Calgary, 1403-29th Street NW, Calgary, Alberta, T2N 2T9, Canada; Ichan School of Medicine at Mount Sinai, UNITED STATES

## Abstract

Tregs infusion reverts proteinuria and reduces renal lesions in most animal models of nephrotic syndrome (i.e. Buffalo/Mna, Adriamycin, Promycin, LPS). IL2 up-regulates Tregs and may be an alternative to cell-therapy in this setting. To evaluate a potential role of IL2 as Tregs inducer and proteinuria lowering agent in human nephrotic syndrome we treated 5 nephrotic patients with 6 monthly cycles of low-dose IL2 (1x10^6^ U/m^2^ first month, 1.5x10^6^ U/m^2^ following months). The study cohort consisted of 5 children (all boys, 11–17 years) resistant to all the available treatments (i.e. steroids, calcineurin inhibitors, mycophenolate, Rituximab). Participants had Focal Segmental Glomerulosclerosis (3 cases) or Minimal Change Nephropathy (2 cases). IL2 was safe in all but one patient who had an acute asthma attack after the first IL2 dose and did not receive further doses. Circulating Tregs were stably increased (>10%) during the whole study period in 2 cases while were only partially modified in the other two children who started with very low levels and partially responded to single IL2 Proteinuria and renal function were not modified by IL2 at any phase of the study. We concluded that low-dose IL2 given in monthly pulses is safe and modifies the levels of circulating Tregs. This drug may not be able to lower proteinuria or affect renal function in children with idiopathic nephrotic syndrome. We were unable to reproduce in humans the effects of IL2 described in rats and mice reducing *de facto* the interest on this drug in nephrotic syndrome.

**Trial Registration:**

ClinicalTrials.gov NCT02455908

## Introduction

Nephrotic syndrome unresponsive to drugs remains an unresolved and clinically relevant problem.[1] Lack of available therapies, progression to chronic renal failure and recurrence after renal transplant are critical issues that need to be address by basic science research and require new drug development.[2]

Experimental models of nephrotic syndrome suggest a multistep pathogenesis in which there is an equilibrium between immunological stimuli and counter-balancing regulatory mechanisms mainly involving.[3] The implication of the innate immune system is evident in mice treated with lipopolysaccharide (LPS) that develop a transient proteinuria resembling minimal change lesions in humans. LPS up-regulates the expression in podocytes of the co-stimulatory molecule CD80 through Toll Like receptor 4 (TLR-4) signalling independent from T and B cells[4]. In human beings, increased urinary levels of CD80[5] have been shown during the active phase of minimal change nephropathy and its inhibition by abatacept, a fusion CTLA4-Ig molecule, reduces proteinuria in some patients with post-transplant recurrence of focal segmental glomerulosclerosis.[6, 7] Also oxidants may have a role at this step. Their implication is indirectly supported by the renal toxic effects of adriamycin and puromicin aminonucleoside, two compounds metabolized by xantine oxidase through the hypoxantine pathway implying oxidant formation.[8–10]. When given to rats, both adriamycin and puromicin cause proteinuria and histological lesions of minimal change lesions evolving to glomerulosclerois similar to the human condition.

Tregs implication has been shown in the same and in other experimental models of nephrosis: post-trasplant proteinuria and regression of the nephropathy was obtained by infusion of Tregs in Buffalo/Mna rats that spontaneously develop glomerulosclerosis[11] and in rats with adriamycin nephrosis, in which case Tregs were directly infused[12] or stimulated by adenosine. Tregs are known to secrete CTLA-4, which binds CD80 and block the co-stimulatory pathway of activation of T cells.[13, 14] Blockage of the co-stimulatory pathway and modulation of pro- and anti-inflammatory compounds link Tregs to the innate immunity and may explain why they are protective in animal models of nephrosis. [12][13, 14]

Using IL2 is a practical alternative to direct infusion of Tregs. In fact, this cytokine stimulates Tregs maturation from T progenitor and is currently considered a potential drug in clinical conditions in which high Tregs may be beneficial to the outcome. Infusion of IL2 has been shown to increase Tregs in the circulation and in tissues of mice with LPS experimental nephrosis.[15, 16] Low-dose IL2 has also been successfully utilized in human beings with HCV-induced vasculitis[17] and in patients with Graft-versus-Host disease[18] to increase circulating Tregs and improving these conditions. Low dose IL2 (1–3 million IU per m^2^ per day roughly equivalent to 1:10 of the posology utilized for cancer) is free of relevant side effects and therefore its use may be extended to other pathologic conditions. Expansion of Tregs by low dose-IL2 could represent an alternative to cell therapy with Tregs infusion in patients with nephrotic syndrome refractory to all other treatments (i.e. steroids, calcineurin inhibitors, anti-CD20 monoclonal antibodies). An open-label case-control phase 1–2 pilot trial was designed to assess safety and clinical and immunologic effects of repeated administration of recombinant low dose IL2 in 5 patients with idiopathic nephrotic syndrome unresponsive to all treatments used for this condition.

## Results

**Fig 1:**
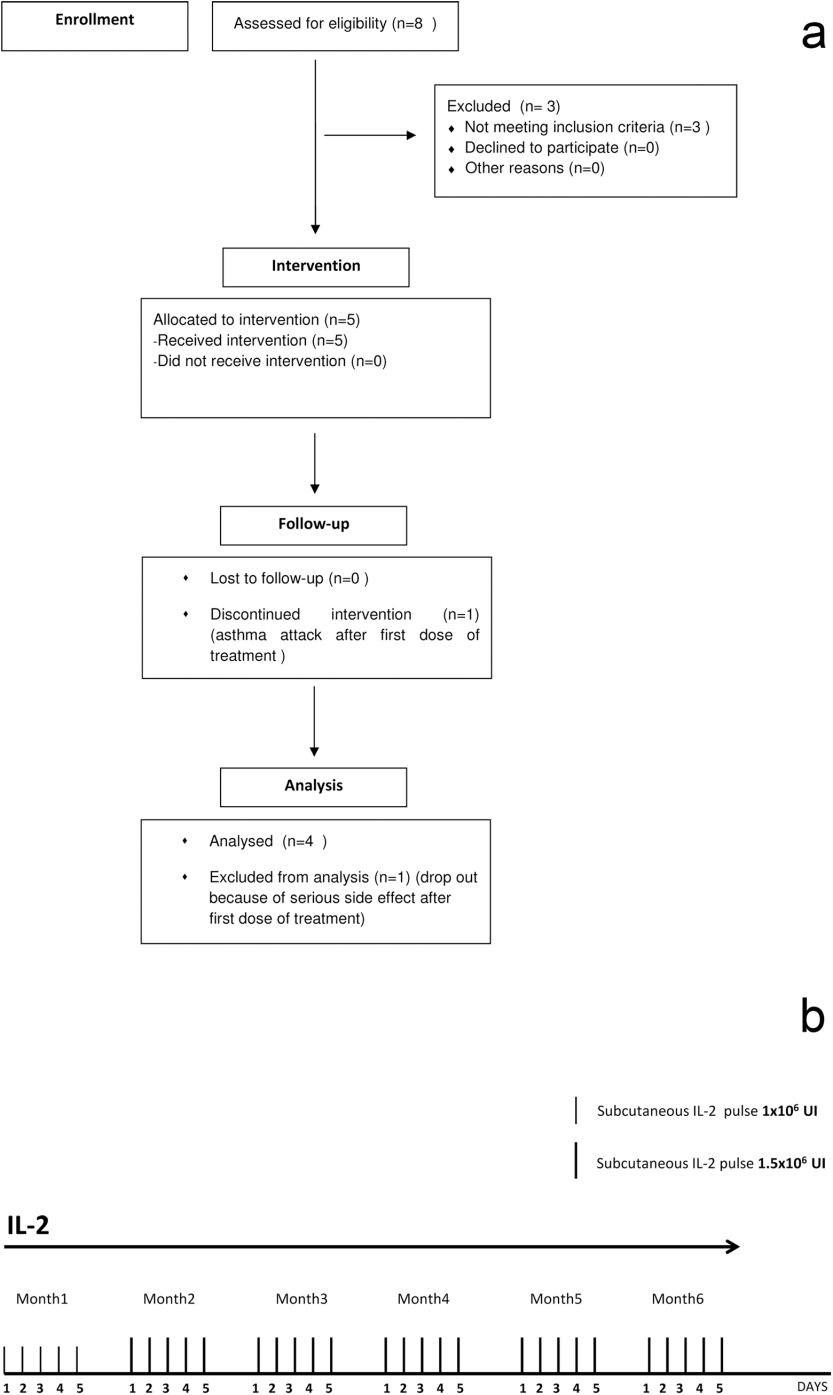
Flow Chart and scheme for IL2 administration. **(a)** All 5 children were allocated to intervention; one (Pt 5) was excluded from the study due to a severe adverse event (asthma attack after the first IL-2 Infusion). All remaining participants remained in the study for at least 180 days. **(b)** All patients received the first IL2 dose of 1x10^6^ U/m^2^ (subcutaneous recombinant IL2 (Proleukin®) that was repeated for additional 4 days in 4 children for the first cycle (month one). These 4 children continued with other 5 cycles of IL2 1.5x10^6^ U/m^2^ per day for 5 days at the beginning of the following 5 months.

Between February and July 2012, 5 children with long lasting nephrotic syndrome unresponsive to drugs were enrolled in the study (**Fig 1A**). All had normal or borderline renal function. FSGS and minimal change disease were the underlying pathology; molecular sequencing of the major genes responsible for recessive forms of nephrotic syndrome were negative. In the years preceding IL2, patients had been unsuccessfully treated with steroids, calcineurin inhibitors, Rituximab and in some cases with plasmapheresis **(Table 1).** Patients were treated according to the scheme shown **Fig 1B.** This represents a minimal modification of the original protocol utilized by Saadoun et al.[19] in adults with vasculitis that were treated with a standard dose of IL2 3 x10^6^ not modified for the body surface area. The timing of IL2 infusions and the number of treatments were modified as well in consideration of the chronic nature of nephrotic syndrome unresponsive to drugs. Overall, the cumulative dose utilized in this study was higher than previous reports.

**Table 1:** Clinical characteristics and laboratory data of patients at the start of IL2 and therapeutical history.

Patient N.	1	2	3	4	5
**Sex**	M	M	M	M	M
**Age (yrs)**	17	11	15	17	15
**Age at Onset (yrs)**	13	2	10	3	13
**Histology**	FSGS	FSGS	MCD	FSGS	MCD
**Previous Therapy**	CsA,FK,RTX,PEX	CsA, FK, RTX	CsA, FK, RTX	CYC, CsA,FK,RTX	CsA, FK,RTX,PEX
**N. of RTX**	1	2	3	2	3
**Last RTX (months)**	27	24	34	5	39
**Current therapy**	ARB	ACE-I	PDN,CsA, ACE-I	ACE-I	ACE-I
**sCreatinine mg%**	1	1,8	1,2	1,1	1,5
**sAlbumin gr%**	3,1	2,8	1,8	3,4	3,2
**uProt Gr/24hr**	5,1	5,6	2,4	2,3	3,5

CsA = Cyclosporine, FK = Tacrolimus, RTX = Rituximab, PEX = plasma Exchange, CYC = Cyclophosphamide, PDN = prednisone (low dose).

**Fig 2:**
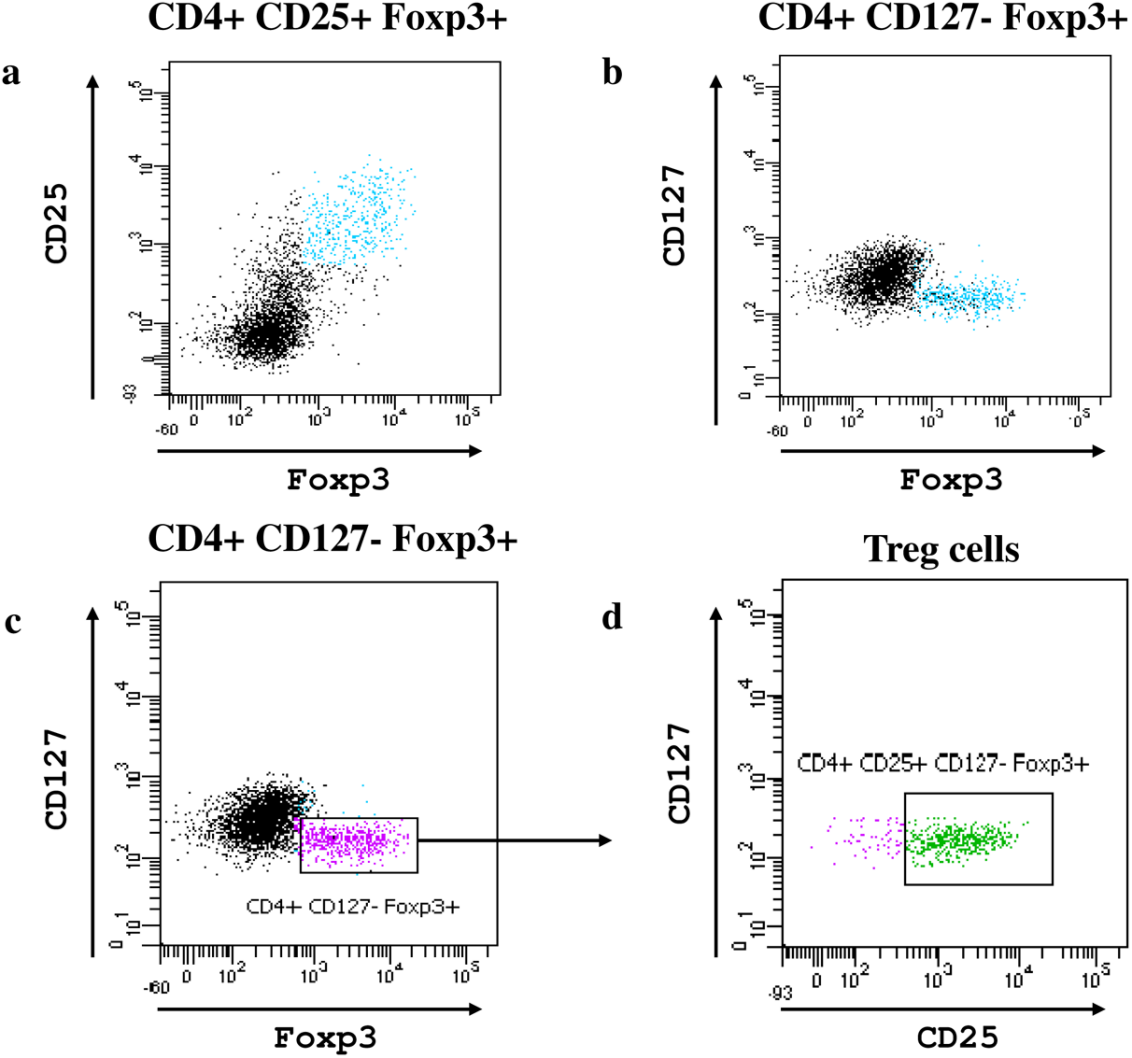
FACS plot. Example of Tregs characterization and gating strategy: CD4+ lymphocytes obtained for peripheral blood were stained with anti-CD25-PE, Foxp3-APC and CD127-FITC; co-expression of different fluorochomes are shown in **a)** CD25+ Foxp3+, **b)** CD127- Foxp3+, **c)** the same as in **b** and **d)** CD4+ CD25+ CD127- Foxp3+ [20].

Tregs were evaluated in peripheral heparinised blood following the procedure shown in **Fig 2.**

**Fig 3:**
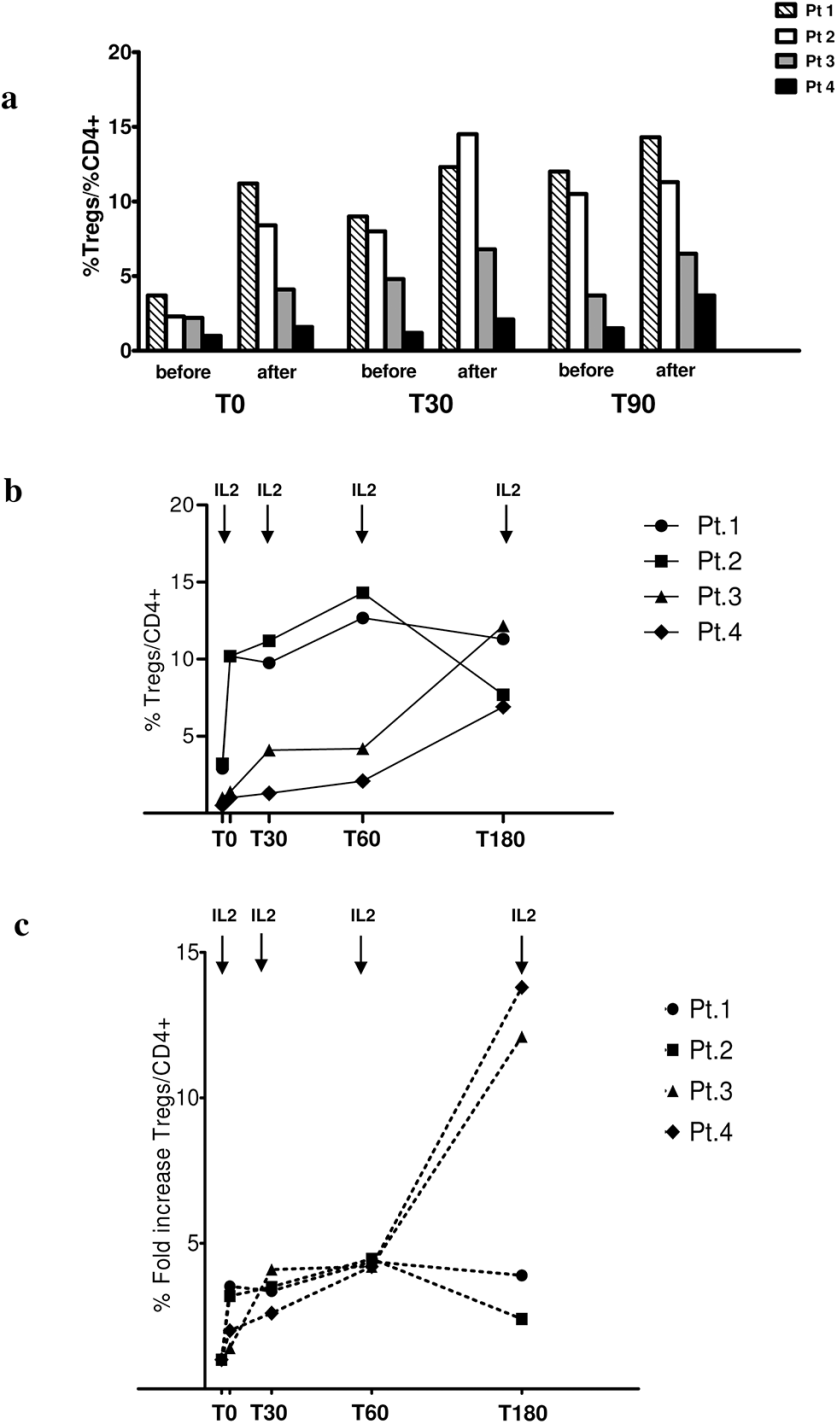
Tregs variation in the mid and long-terms. Levels of circulating Tregs were evaluated every month before IL2 and at the 5^th^ IL2 infusion. Results were expressed as total percentage **(b)** or as fold increment in respect to T0 **(c).** Variations during the single IL2 course (i.e. Tregs levels at day 1 and 5) are reported in **(a)**.

Tregs levels are shown in **Fig 3B and 3C**: they were determined at the pre-treatment (T0) and during the treatment phases (T+30 days, T+90 days and T+180 days) in all cases at the beginning of the IL2 cycle and after the 5^th^ infusion. There were notable variations of Tregs levels within the 5 days of IL2 infusion in 3 patients whereas one child who started from the lowest value presented only minimal changes initially and responded only after the 3^rd^ cycle of IL2 (Fig 3B and **3**C). During the whole study period Treg levels increased by approximately 10% with differences in rapidity of response from 30 days for patient 1 and 2, up to 150 days for patient 4 (Fig 3B). This behaviour is given as fold increment of Tregs at various periods in Fig 3C.

**Fig 4:**
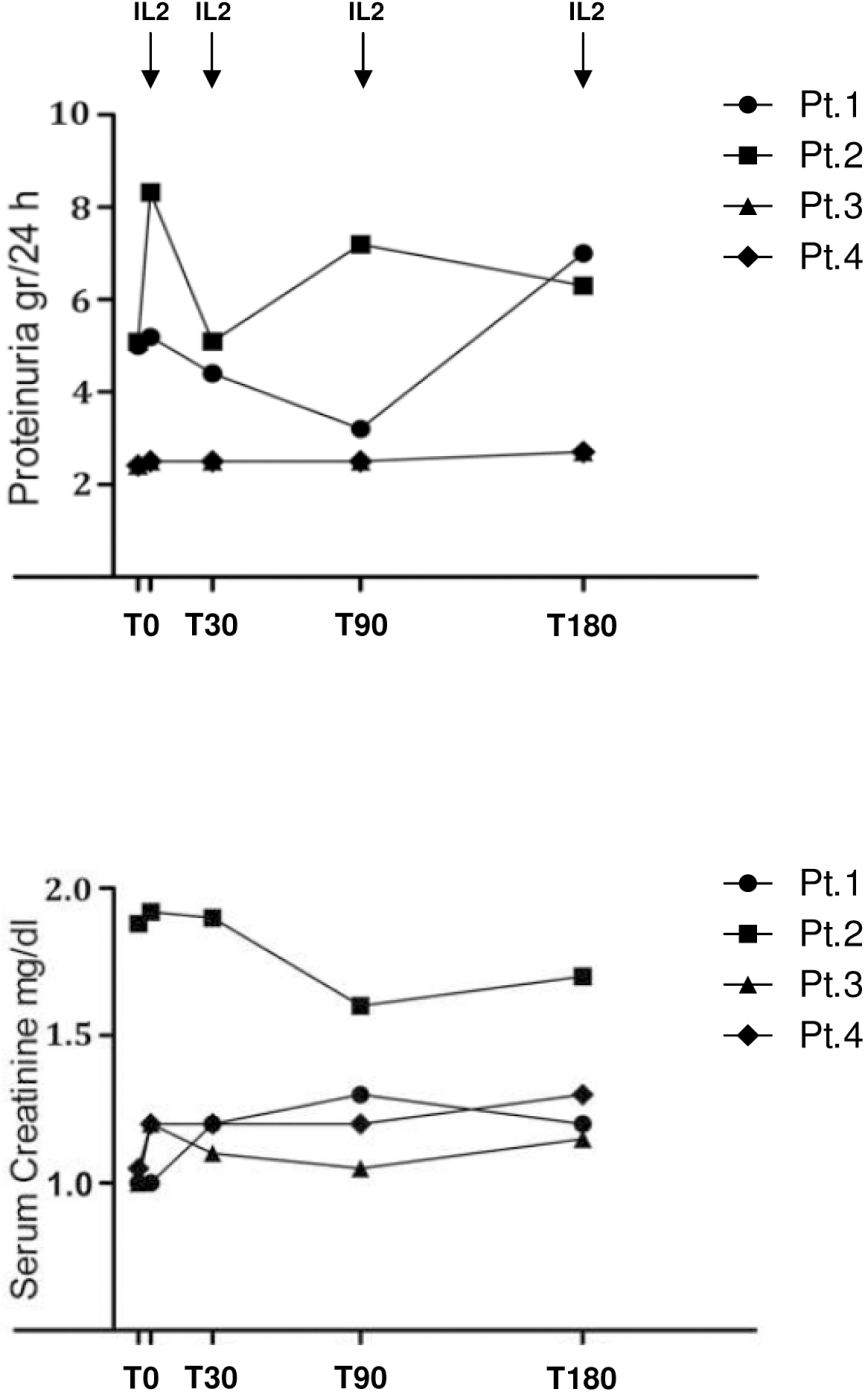
Clinical parameter outcome during treatment. Proteinuria and serum creatinine outcome in patients treated with low-dose IL2 for 6 months with monthly cycles of 5 subcutaneous infusions. Arrows indicate timing of IL2 administration.

Proteinuria did not change during the study period (**Fig 4**). Similarly, serum protein concentration remained low and serum creatinine levels remained essentially the same (**Fig 4**). After 2 years from the study end date all patients had kidney disease progression requiring peritoneal or hemo-dialysis.

### Adverse events

One patients (pt 5) dropped-out from the study for an asthma attack after the first dose of IL2 requiring interruption of treatment. Bronchospasm started 6 hours after the first IL2 and required therapy with salbutamol and steroids for 48 hours. During this episode eosinophils count was 1%. This patient had presented a few mild asthma attacks several years before that required specific therapy (salbutamol and steroids) for brief periods. No relevant side effects were noted in the other patients during the whole period with the exception of transient fever typically starting 2–4 hours after injection and remitting after paracetamol. Laboratory parameters (blood cell counts, transaminases, LDH, serum iones, RCP) remained stable.

## Discussion

Animal models of nephrotic syndrome suggest that Tregs may be utilized in therapy of nephrotic syndrome. Le Berre and *col*.[11] infused Tregs in Buffalo/Mna rats that spontaneously develop glomerulosclerosis obtaining reduction of pre- and post-transplant proteinuria and regression of the nephropathy. Wang and *col*. [12] reported the same protective role of Tregs in adriamycin nephrosis that was obtained by direct infusion of Foxp3-transduced T cells.

IL2 is considered an alternative to cell-therapy since this cytokine plays a key up-regulatory effect on Tregs. The administration of low doses IL2 coupled with anti-IL2 antibody is of interest since it induces a selective increment of those cells responsible for the generation of Tregs (activated CD4^+^ cells/Tconv).[21, 22] Polhill and *col*.[15] infused IL2/IL2-Ab in rats with adriamycin nephrosis and induced Treg expansion with documented improvement of renal function and reduced inflammation. Bertelli and *col*.[16] recently published more controversial and articulated results. They compared the effects of IL2 alone or combined with IL2-Ab in mice with LPS nephropathy showing superiority of IL2 alone to reduce proteinuria and renal damage. While the combination of IL2 and IL2Ab administration enhanced peripheral and tissue Treg levels more than IL2 alone, IL2 alone resulted in superior protection against proteinuria. This lack of direct correlation between Tregs levels and renal protection partially contradicts the direct role of Tregs in reducing proteinuria and supports a more complex mechanism potentially involving innate immunity.

We utilized IL2 alone to attempt a direct approach in human beings based on the above encouraging results in mice. Doses and phases of drug administration were chosen on the basis of the recent reports from two independent groups on the use of low-dose IL2 for HCV renal vasculitis[17] and in patients with GVHD[18]. Results from both studies were relevant to show safety of low dose IL2. We designed a pilot case-control study in 5 patients with nephrotic syndrome resistant to drugs and included patients with borderline glomerular filtration rate values. We treated these children following the original protocol utilized by Saadoun et al[19] in adults with vasculitis and modifying the dose based on the body surface area. Overall, we gave a larger total cumulative dose compared to the original protocol (i.e. 60.5 x 10^6^ vs.52.5 x 10^6^). The laboratory approach to Treg quantitation considered most recent evidence in human beings on cell population more sensitive to IL2 that have been identified as CD4+ CD25+ FOXP3+CD127-[20]. The later markers allow to exclude Teff contamination thus leading to more reliable quantitation of Treg levels. The trend to a stable increase of Treg count in all patients during the whole period confirmed the accuracy of this protocol. The increase in Treg levels we obtained however did not result in reduction in urinary proteinuria indicating that IL2 does not modify the course of proteinuria in patients with nephrotic syndrome resistant to drugs in spite an evident increment of circulating Tregs.

In other studies[17, 18] comparable IL2 doses were given more frequently (i.e., every 15 days as opposed to monthly) achieving earlier peak of Treg count (i.e., only after 38 days) but was followed by levels 50% lower in the following 3 months. More frequent infusions may impact compliance of children and their families and determine serious problems correlated with feasibility of infusions since the drug is instable, it must be prepared every day in a specialized structure and infused within few hours after preparation. Fever attacks (and potentially asthma) are strictly dependent on dose. At the dose above, the treatment was safe and did not modify any clinical and laboratories parameters; it was well tolerated in 4 patients who required minimal ancillary therapies with paracetamol and anti-histaminic to reduce transient bouts of fever occurring after 2 hours from infusion. One child presented, instead, an acute asthma attack after the first IL2 dose which required specific therapies with steroids and β stimulators.

Our study has limitations, principally due to the non-randomized design and the small sample size. However, this was a pilot study that was not designed to answer an efficacy question with a pre-specified effect. The objective was explorative in nature and principally aimed to detect the existence of a signal to be tested in a clinical trial. The second limitation is relative to the advanced state of renal disease that may have reduced the potential effects of IL2 therapy. Further studies may be justified in children with shorter disease duration who fail to respond to available recommended therapies.

## Materials and Methods

This study was approved by local Ethics Committee, "Committee for Proper Use of Drugs, Ligurian Ethics Committee" on February, 22nd, 2012. A written informed consent was in all cases obtained from parents of patients. All clinical investigation have been conducted according to the principles expressed in the Declaration of Helsinki. This study was not registered before enrolment of participants, because of the “off label drug use” design of the trial.

Actually, the trial has been registered in Clinicaltrials.gov, registration number NCT02455908.

For editorial reason we now confirm that all ongoing and related trials for this drug are registered.

### Patients

Between June 2011 and January 2012 we screened in the outpatient nephrology of the IRCCS Giannina Gaslini Children Hospital 8 children affected by drug resistant idiopathic nephrotic syndrome who already presented initial renal function impairment (Table 1). Negativity to molecular genetics tests relative to the 3 genes (NPHS1, NPHS2, WT1) whose mutations are known to be responsible of almost 80% of familiar cases were requested for enrollment. Of these, 5 male children were recruited after having documented the persistence of proteinuria for at least 1 year (median 5, range 2–14 yrs), during which they had received a combination of steroids, cyclosporine/tacrolimus, mycophenolate mofetil and Rituximab. The aim of the study was to assess safety and clinical and immunologic effects of repeated administration of recombinant low dose IL2.

Definition of drug resistance was based on consolidated criteria according to which proteinuria in nephrotic range persisted after a cycle of steroids of at least 3 months and an association with cyclosporine/tacrolimus for at least other 6 months. At the time of enrolment all patients had also received 1–3 doses Rituximab (**Table 1**) [23, 24].

Renal histology showed FSGS in 3 patients and minimal change disease in 2 patients. At the time of enrolment all patients were receiving ACEi or ARB.

Patients data collection, treatment administration, molecular genetics and data analysis were all performed at Division of Nephrology, Dialysis, Transplantation and Laboratory on Physiopathology of Uremia, Giannina Gaslini Children Hospital, Genoa, Italy.

### Treatment

Recombinant IL2 (Proleukin®) was given subcutaneously with single daily doses given for 5 days, at one month interval for 6 months following the protocol developed for renal vasculitis (see scheme in **Fig 1B**). In the former cycle it was utilized IL2 1x10^6^ /m^2^, starting from month 2 the dose of IL2 was increased to 1.5 x10^6^ /m^2^. The drug was given according to pre-specified protocol (S1 and S2 Files).

### Primary outcome

The primary outcome of the study was the achievement of complete or partial disease remission after a complete treatment. Complete remission was defined as urinary levels of proteinuria <0.3 g/day for 3 consecutive days. Partial remission was defined as proteinuria reduction of 50% or greater from the presenting value for 3 consecutive days.

### Secondary outcomes

Secondary outcomes of the study were variations in Tregs and creatinine levels after IL-2 administration. Tregs levels were determined at the pre-treatment phase (T0) and during the treatment phases (T+30 days, T+90 days and T+180 days) in all cases at the beginning of the IL2 cycle. Creatinine levels were determined monthly.

### Tregs determination

Heparinized peripheral blood obtained from patients was layered onto a Ficoll-Histopaque 1077 (Sigma-Aldrich, St. Louis, MO, USA) density gradient and centrifuged at 2,000 rpm for 30 minutes. Mononuclear cells were collected at the interfaces, washed and re-suspended in Phosphate Buffer Saline (PBS), supplemented with 2mM EDTA, and further enriched with CD4+ lymphocytes by negative selection using an appropriate CD4+ T cells isolation kit (Miltenyi Biotec, Bergish Gladbach, Germany).

For Tregs evaluation, CD4+ lymphocytes were stained with an Antibody cocktail containing CD4+ PerCP Cy5.5, CD25 PE and CD127 FITC (all from BD Biosciences Pharmingen, San Diego, CA, USA), for 20 minutes at room temperature, and subsequently fixed and permeabilized for intranuclear FoxP3 expression with an anti-human Foxp3-APC staining set (eBioscience, San Diego, CA, USA), accordingly to manifacturer’s instructions. Acquisitions were made on BD-FACSCanto II Flow Cytometer, equipped with FACS Diva Software; Treg cells were identified in the CD4+ CD25 high, CD127^_^ Foxp3+ cells fraction and expressed as percentage of total CD4+ lymphocytes.

## Supporting Information

S1 File -Protocol of the study *English* version. Protocol for low-dose IL2 in nephrotic patients pilot study.
(PDF)

S2 File Protocol of the study *Italian* version. Protocol for low-dose IL2 in nephrotic patients pilot study.
(PDF)

S3 File Trend Statement checklist. (PDF)
