# Supplementary material for: A Pilot Study of IL2 in Drug-Resistant Idiopathic Nephrotic Syndrome
Source: PLoS One. 2015 Sep 28;10(9):e0138343. doi: 10.1371/journal.pone.0138343 (PMC4587361; doi:10.1371/journal.pone.0138343)
Supplement: S1 File — Protocol for low-dose IL2 in nephrotic patients pilot study. (PDF) [file pone.0138343.s001.pdf]

Istituto Giannina Gaslini

U.O. di Nefrologia e Dialisi

Responsabile.: Dott. Gian Marco Ghiggeri

Largo G. Gaslini, 5 – 16147 Genova

Tel. 010-5636 276

Fax 010-395214

e-mail: nefrologia@ospedale-gaslini.ge.it

Use of IL-2 (Proleukin) in drug resistant idiopathic nephrotic syndrome.

Idiopathic nephrotic syndrome with pediatric onset is a well-characterized entity which includes separate pathological patterns, potentially due to different mechanisms. Steroids constitute the first therapeutical approach and are usually utilized in the acute phase of the disease, due to their power to induce clinical remission.

However, in some cases characterized by steroid resistance, it is necessary to start a combined therapy with calcineurin inhibitor, with the risk of development of resistance to the pharmacological association. Treatment of steroid resistant cases remain an unsolved problem, not only in term of therapeutical strategy; indeed, it is necessary to define the impact of drug resistance in the evolution of renal damage toward degenerative forms of renal sclerosis.

Retrospective data obtained from large cohort studies suggest a close association between drug resistance and progression to chronic renal failure.

Rituximab has been recently introduced in clinical practice of idiopathic nephrotic syndrome, modifying clinical outcome of steroid-dependent patients, without effects in multi drug resistant cases.

The therapeutical failure in many patients is probably caused by a not well defined pathogenesis of idiopathic nephrotic syndrome: although recent findings in familiar forms suggest a role of genetic alterations of podocyte's proteins in some patients, many evidences point to the hypothesis of a dysregulation in the mechanisms underlying innate immunity as a putative pathogenic factor. Oxygen radicals seem to play a key role as mediators of innate immunity response, that also seems to be hampered by T regulatory cells (Tregs) [1-4]. Data from experimental models strongly support a role of a defective Tregs activity in generation of proteinuria, which is reduced in many cases by the use of cellular therapies with conditioned Tregs[1].

Recent clinical data showed an effect of low doses of IL-2 in inducing a stable increase in peripheral Tregs levels (+400%), which is associated, in some clinical conditions (i.e. crioglobulinemic vasculitis), with an important clinical improvement (reduction of proteinuria, improvement of renal function). No significant side effects were observed until now.

These data, together with what we above said about the pathogenesis of idiopathic nephrotic syndrome suggest a possible clinical effect of IL-2 in this pathology, inducing to a therapeutic attempt.

Therapeutical treatment with IL2 in idiopathic nephrotic syndrome.

We propose to utilize low doses of IL2 (Proleukin) in patients with drug resistant (steroids, calcineurin inhibitors, Rituximab) idiopathic nephrotic syndrome, following the therapeutical scheme indicated for crioglobulinemic nephropathy:

cycle1: IL2 $1x10^6$ /m^2 s.c for 5 consecutive days

cycle2: IL2 1.5 $x10^6$ / m^2 s.c for 5 consecutive days, starting from 3 weeks after the first cycle.

cycle3: IL2 1.5 $x10^6$ /m2 s.c for 5 consecutive days, starting from 6 weeks after the first cycle.

cycle 4: IL2 1.5 $x10^6$ /m2 s.c for 5 consecutive days, starting from 9 weeks after the first cycle.

Current therapy with steroids and calcineurin inhibitors (Prograf) will be maintained during the first cycle and progressively reduced during the subsequent cycles.

The first cycle will be performed during hospitalization in our Unit; subsequent cycles will be performed at nephrology outpatients. All laboratory values normally utilized in the follow up of patients affected by idiopathic nephrotic syndrome will be evaluated during the first week of treatment and at the end of the protocol, together with specific cellular values (Tregs, B cells, NK).

Tests

Cycle T0-5 CD127-CD25+CD4+FOXP3/ CD127-CD25-CD4+ /NK (6 complete blood count tubes, C Trasf.)

Cycle2 T0-5 CD127-CD25+CD4+FOXP3/ CD127-CD25-CD4+ /NK (,, ,, ,,)

Suppression tests

Common blood (complete blood count, renal function, electrophoresis, PTH) and urinary tests (urinalysis, dosage of proteinuria on 24 hours urine collection) will be performed monthly.

References

1- Le Berre L, Bruneau S, Naulet J, Renaudin K, Buzelin F, Usal C, Smit H, Condamine T, Soulillou JP, Dantal J. Induction of T regulatory cells attenuates idiopathic nephrotic syndrome.***J Am Soc Nephrol. 2009 Jan;20(1):57-67***.

2- Bertelli R, Trivelli A, Magnasco A, Cioni M, Bodria M, Carrea A, Montobbio G, Barbano G, Ghiggeri GM. Failure of regulation results in an amplified oxidation burst by neutrophils in children with primary nephrotic syndrome. ***Clin Exp Immunol 2010 Jul;161(1):151-158***

3-Bertelli R, Bodria M, Nobile M, Alloisio S, Barbieri R, Montobbio G, Patrone P, Ghiggeri GM. Regulation of innate immunity by the nucleotide pathway in children with idiopathic nephrotic syndrome. ***Clin Exp Immunol 2011 Oct;166(1):55-63***

4- Saadoun D, Rosenzwajg M, Joly F, Six A, Carrat F, Thibault V, Sene D, Cacoub P, Klatzmann D. Regulatory T-cell responses to low-dose interleukin-2 in HCV-induced vasculitis. ***NEJM 2011 Dec 1;365(22):2067-77***
