# Supplementary material for: A Pilot Study of IL2 in Drug-Resistant Idiopathic Nephrotic Syndrome
Source: PLoS One. 2015 Sep 28;10(9):e0138343. doi: 10.1371/journal.pone.0138343 (PMC4587361; doi:10.1371/journal.pone.0138343)
Supplement: S2 File — Protocol for low-dose IL2 in nephrotic patients pilot study. (PDF) [file pone.0138343.s002.pdf]

Istituto Giannina Gaslini
U.O. di Nefrologia e Dialisi
Responsabile.: Dott. Gian Marco Ghiggeri
Largo G. Gaslini, 5 – 16147 Genova
Tel. 010-5636 276
Fax 010-395214
e-mail: nefrologia@ospedale-gaslini.ge.it

Proposta di utilizzo di IL2 (Proleukin) nella sindrome nefrosica idiopatica resistente ai farmaci.

Background

La sindrome nefrosica idiopatica ad insorgenza in età pediatrica è un'entità ben caratterizzata nella sua fenomenologia clinica pur raccogliendo entità patologiche separate e potenzialmente dovute a meccanismi diversi. I corticosteroidi costituiscono il primo approccio terapeutico alla malattia e vengono di norma utilizzati nel trattamento della fase acuta in quanto potenzialmente efficaci nell'indurne la remissione clinica. Esistono però casi con resistenza agli steroidi che richiedono una terapia combinata con inibitori delle calcineurine e che possono anche sviluppare una resistenza all'associazione. L'approccio terapeutico nei casi con resistenza ai farmaci resta un contingenza clinica non risolta. Oltre al problema clinico legato alla gestione della sindrome nefrosica acuta resta da definire l'impatto che la farmaco resistenza può giocare nell'evoluzione del danno renale verso patologie degenerative con sclerosi renale. Dati retrospettivi della letteratura derivanti da studi collaborativi in coorti sufficientemente numerose suggeriscono la stretta associazione della farmaco resistenza con la progressione verso l'insufficienza renale cronica. Recentemente è entrato nella pratica clinica della sindrome nefrosica idiopatica il Rituximab che ha di fatto modificato la storia clinica di quella parte di pazienti che avevano precedentemente dimostrato una dipendenza all'uso combinato di calcineurine e steroidi ma non ha migliorato la sensibilità nei pazienti con resistenze multiple.

La ragione degli insuccessi terapeutici in una parte di pazienti è chiaramente dovuta al fatto che di molto si ignora sulla patogenesi della malattia. Nonostante i rilevanti avanzamenti dell'ultimo decennio relativi alle forme di sindrome nefrosica famigliare abbiano dimostrato il coinvolgimento di geni/proteine strutturali podocitarie in una parte di pazienti, resta l'ipotesi di un coinvolgimento dell'immunità innata e della sua regolazione nella patogenesi della malattia. Sembrano avere un ruolo determinante mediatori della risposta immune innata, quali i radicali dell'ossigeno ed è ipotizzata una alterazione della regolazione di essa da parte di Treg[1-4]. Dati nei modelli sperimentali della malattia supportano fortemente l'implicazione di un difetto legato ai Treg nella genesi della proteinuria che viene ridotta ed in molti casi risposta con l'uso di terapie cellulari con Treg condizionati[1].

Recenti esperienze cliniche[4] hanno dimostrato che basse dosi di IL2, ripetute nel tempo, inducono un stabile aumento dei livelli di Treg (+400%) e si associano, in situazioni cliniche studiate (i.e.

vasculite criobglobulinemica con nefrite), a miglioramento clinico importante (riduzione della proteinuria, miglioramento della funzione renale). Non sono stati osservati effetti collaterali degni di nota.
Tale dato, unitamente a quanto riferito circa la patogenesi della sindrome nefrosica idiopatica, fa prospettare un effetto clinico importante della IL2 in questa patologia e ne suggerisce un tentativo terapeutico.

Programma di terapia con IL2 nella sindrome nefrosica idiopatica.
Si propone di utilizzare basse dosi di IL2 (Proleukin) in pazienti con sindrome nefrosica idiopatica resistente ai farmaci(steroidi, inibitori delle calcineurine, Rituximab) utilizzando lo stesso schema a basse dosi già riportato per la nefropatia crioglobulinemica:
ciclo1: IL2 $1x10^{6}$ /m2 s.c per 5 giorni continuativi
ciclo2: IL2 1.5 $x10^{6}$ / m2 s.c per 5 giorni continuativi dopo 3 settimane dal 1 ciclo
ciclo3: IL2 1.5 $x10^{6}$ /m2 s.c per 5 giorni continuativi dopo 6 settimane dal 1 ciclo
ciclo4: IL2 1.5 $x10^{6}$ /m2 s.c per 5 giorni continuativi dopo 9 settimane dal 1 ciclo

la terapia ordinaria attualmente con Deltacortene e Prograf sarà mantenuta durante il primo ciclo e sarà poi ridotta nei cicli successivi.

Il primo ciclo sarà effettuato in regime di ricovero ordinario, i cicli successivi in regime di follow-up ambulatoriale. Si valuteranno durante la prima settimana ed a conclusione dei cicli di infusione i parametri laboratoristici generali utilizzati nei pazienti con sindrome nefrosica; si valuteranno i parametri cellulari specifici (Treg, Bcells, NK).

Tests
Ciclo1 T0-5 CD127-CD25+CD4+FOXP3/ CD127-CD25-CD4+ /NK (6 provette emocromo, C Trasf.)

Ciclo2 T0-5 CD127-CD25+CD4+FOXP3/ CD127-CD25-CD4+ /NK (,, ,, ,,)
Test di soppressione

Comuni valutazioni seriche (emocromo, creatinina/azoto, ioni, proteine elettroforesi, PTH) ed urinarie (esame urine, proteinuria 24h) mensilmente

Referenze:

1- Le Berre L, Bruneau S, Naulet J, Renaudin K, Buzelin F, Usal C, Smit H, Condamine T, Soulillou JP, Dantal J. Induction of T regulatory cells attenuates idiopathic nephrotic syndrome.

J Am Soc Nephrol. 2009 Jan;20(1):57-67.

2- Bertelli R, Trivelli A, Magnasco A, Cioni M, Bodria M, Carrea A, Montobbio G, Barbano G, Ghiggeri GM. Failure of regulation results in an amplified oxidation burst by neutrophils in children with primary nephrotic syndrome.
Clin Exp Immunol 2010 Jul;161(1):151-158

3-Bertelli R, Bodria M, Nobile M, Alloisio S, Barbieri R, Montobbio G, Patrone P, Ghiggeri GM. Regulation of innate immunity by the nucleotide pathway in children with idiopathic nephrotic syndrome.
Clin Exp Immunol 2011 Oct;166(1):55-63

4- Saadoun D, Rosenzwajg M, Joly F, Six A, Carrat F, Thibault V, Sene D, Cacoub P, Klatzmann D. Regulatory T-cell responses to low-dose interleukin-2 in HCV-induced vasculitis.
NEJM 2011 Dec 1;365(22):2067-77
